# Outcomes of Regenerative Endodontic Therapy Using Dehydrated Human-Derived Amnion–Chorion Membranes and Collagen Matrices: A Retrospective Analysis

**DOI:** 10.3390/biomimetics10080530

**Published:** 2025-08-13

**Authors:** Anjali K. Dave, Julia Y. Cheung, Sahng G. Kim

**Affiliations:** Division of Endodontics, Columbia University College of Dental Medicine, New York, NY 10032, USA; anjalidave26@gmail.com (A.K.D.); jcheungdmd@gmail.com (J.Y.C.)

**Keywords:** outcomes, amnion–chorion membrane, collagen matrix, bioactive scaffolds, pulp regeneration, regenerative endodontics, biomaterials

## Abstract

Dehydrated human-derived amnion–chorion membranes (ACM), known for their bioactive composition of growth factors and cytokines, have demonstrated potential as a bioactive scaffold in regenerative medicine; however, their clinical application in regenerative endodontic procedures (REPs) remains unexplored. This retrospective study aimed to evaluate the clinical and radiographic outcomes of REPs using ACM compared to collagen matrices (CM) in immature necrotic permanent teeth. Forty-one immature necrotic teeth from 38 patients (mean age: 14.68 ± 7.43 years) were treated with REPs using either ACM (*n* = 21) or CM (*n* = 20) scaffolds over a mean follow-up period of 23.23 months. Outcomes assessed included survival, success, root development measured by radiographic root area (RRA), and pulp sensibility. Independent t-tests compared outcomes between groups, while Cox regression and generalized linear models identified predictors of treatment outcomes. Overall survival and success rates were 87.8% and 82.9%, respectively. ACM-treated teeth achieved 90.5% survival and 85.7% success rates, while CM-treated teeth demonstrated 85.0% survival and 80.0% success rates, with no statistically significant differences between groups (*p* > 0.05). Root development occurred in 85.4% of cases overall, with significant RRA increases of 13.89 ± 13.95% for ACM and 11.24 ± 11.21% for CM (*p* < 0.05 within each group). Pulp sensibility recovery was observed in 51.2% of treated teeth overall, with 42.9% for ACM-treated teeth and 55.0% for CM-treated teeth (*p* > 0.05). Notably, ACM-treated teeth demonstrated earlier sensibility recovery compared to those of CM-treated teeth. Age was identified as a significant negative predictor of root development outcomes (*p* < 0.05). This clinical study demonstrates that both ACM and CM are clinically effective scaffolds for REPs, achieving high survival rates and promoting root development in immature necrotic teeth. While overall success rates were comparable, ACM showed faster sensibility recovery, suggesting potential biological advantages for enhanced tissue regeneration and earlier functional recovery.

## 1. Introduction

Regenerative endodontic procedures (REPs) have emerged as a biologically driven alternative to traditional root canal therapy, aiming to restore the native structure and function of the pulp–dentin complex rather than replace it with inert materials [1,2,3]. Unlike conventional endodontic treatment, which removes necrotic pulp and seals the root canals, REPs attempt to regenerate a living tissue capable of neurovascular integration and immune surveillance, thereby preserving the long-term vitality of the tooth [4]. Most current REP strategies are cell-free and rely on the induction of apical bleeding to recruit mesenchymal stem cells from the periapical region [2,4]. However, a growing body of research has explored cell homing techniques, which incorporate biomaterial scaffolds to facilitate the migration of stem/progenitor cells and the retention of growth factors within the canal space [5].

The dehydrated human-derived amnion–chorion membranes (ACM) have shown promising regenerative properties due to their rich content of extracellular matrix proteins, cytokines, and growth factors such as vascular endothelial growth factor (VEGF), transforming growth factor-β1 (TGF-β1), and basic fibroblast growth factor (bFGF) [6,7,8,9]. These molecules are known to promote angiogenesis, cell proliferation, differentiation into odontoblast-like cells, and immunomodulation, which are the key biological processes for successful pulp regeneration [5,9]. In addition to their regenerative properties, ACM exhibit antimicrobial activity and low immunogenicity, making them a clinically attractive scaffold material [10,11,12,13,14,15]. ACM have shown efficacy for hard and soft tissue wound healing in regenerative medicine [16,17]. ACM have shown efficacy for hard and soft tissue wound healing in regenerative medicine [16,17]. Their clinical utility extends to regenerative dental procedures, with documented success in alveolar ridge preservation [18], management of periodontal intrabony defects [19], gingival recession [20], furcation involvement [21], and keratinized tissue augmentation around dental implants [22]. Despite the established track record in periodontal regeneration [18,19,20,21,22], clinical evidence on the use of ACM in regenerative endodontics remains limited [9]. Animal and in vitro studies have demonstrated that ACs support fibrous tissue formation, vascularization, and mineralized tissue deposition within the root canals [23,24]. Nevertheless, clinical outcomes in human populations remain unexplored, and no published studies have reported on the use of ACM in REPs for necrotic immature permanent teeth.

Absorbable collagen matrices (CM), commonly used as wound dressings and hemostatic agents, have been widely employed in REPs due to their biocompatibility, ease of clinical handling, and the ability to promote clot formation. However, their therapeutic potential is limited by a lack of endogenous bioactive factors, which are crucial for stimulating cellular recruitment, differentiation, and functional tissue regeneration.

The aim of this study was to evaluate the clinical and radiographic outcomes of REPs using ACM and absorbable CM in immature necrotic teeth. By examining healing of apical periodontitis, root development, and recovery of pulp vitality, this study seeks to provide the first clinical evidence supporting the utility of ACM compared to absorbable CM in regenerative endodontics.

## 2. Materials and Methods

### 2.1. Study Design

This retrospective study was approved by the Institutional Review Board of Columbia University (#AAAS6943) and conducted in accordance with the Declaration of Helsinki. The study was performed at the Division of Endodontics, Columbia University College of Dental Medicine, New York, NY, USA. Clinical and radiographic records of patients who underwent REPs between January 2011 and December 2019 were reviewed. In this study, patients with immature necrotic teeth who received REPs, a follow-up period of at least 6 months, and ACM or CM used as a scaffold material were included. Patients with missing clinical or radiographic data were excluded.

### 2.2. Data Collection

Clinical and radiographic findings were systematically extracted from patient records, including demographic information (age, gender, tooth number), etiology of pulp necrosis, preoperative diagnosis, clinical signs and symptoms, materials used, radiographic evidence of healing, changes in root dimensions, and pulp sensibility testing results.

### 2.3. Regenerative Endodontic Protocol

During the first appointment, local anesthesia was administered, followed by rubber dam isolation and access cavity preparation. The canals were gently irrigated with 1.5–6.0% sodium hypochlorite with minimal or no instrumentation to preserve root dentin structure. After drying with sterile paper points, calcium hydroxide was placed into the canals as an intracanal medicament. The teeth were then sealed with temporary restoration. For cases exhibiting persistent signs and symptoms, the disinfection protocol from the first appointment was repeated.

At the subsequent appointment, teeth were anesthetized with 3% mepivacaine without a vasoconstrictor, reaccessed, and irrigated using 17% EDTA. For the ACM group, a hand file was advanced beyond the apical foramen to evoke bleeding at the apical third of the root canal. ACM (BioXclude, Maxxeus, Kettering, OH, USA) were placed into the canals to interface with apical bleeding. Absorbable CM (CollaPlug, CollaTape, or CollaCote, Zimmer Biomet, Palm Beach Gardens, FL, USA; or HeliPlug, Integra Miltex, Princeton, NJ, USA) were placed over the ACM. In two cases, only ACM were placed without the additional absorbable CM. For the CM group, bleeding was induced to the cementoenamel junction level before placement of the CM (CollaPlug, CollaTape, CollaCote, or HeliPlug) over the formed blood clot.

The canals were sealed with a bioceramic material such as Biodentine (Septodont USA, Lancaster, PA, USA), Endosequence Bioceramic Root Repair Material (Brasseler, Savannah, GA, USA) or ProRoot MTA (Dentsply Tulsa, Johnson, TN, USA) and restored coronally with composite resin, glass ionomer, and resin-modified glass ionomer. Patients were scheduled for a follow-up evaluation after a minimum of 6 months. Clinical and radiographic examinations as well as the responses to pulp sensibility testing were documented.

### 2.4. Radiographic Analysis

Quantification of radiographic root area (RRA) changes was performed using an established protocol [25]. Postoperative and follow-up periapical radiographs were resized to identical dimensions and pixel density. Image alignment and normalization were performed using the TurboReg plugin (Philippe Thevenaz, Biomedical Imaging Group, Ecole polytechnique federale de Lausanne, Lausanne, Switzerland) within ImageJ software (version 1.54; NIH image, Bethesda, MD, USA).

RRA was computed as:

RRA = Total root outline area − Root canal space area

The change in RRA (ΔRRA) and percentage change were derived as:

ΔRRA = Follow-up RRA − Postoperative RRA

% Change in RRA = (ΔRRA/Postoperative RRA) × 100

### 2.5. Outcome Measures and Statistical Analysis

The treatment outcomes were analyzed across three levels. The primary outcome assessed survival and success rates of the treated teeth. Survival was defined as the absence of clinical signs and symptoms with or without radiographic healing status. Success was defined as the absence of clinical signs and symptoms and radiographic evidence of periapical healing (reduction or resolution of periapical radiolucency). Failure was defined as clinical signs and symptoms and/or the radiographic persistence/worsening of periapical lesion (no change or increase in size of periapical radiolucency). The secondary outcome evaluated the root development in immature teeth through measurement of RRA change and quantitative analysis of the RRA percentage change. The tertiary outcome determined the pulp vitality recovery through standard pulp sensibility testing such as cold pulp testing (CPT) or electric pulp testing (EPT).

Demographic data, clinical characteristics, and treatment outcomes including primary, secondary, and tertiary outcomes were summarized using descriptive statistics. Independent t-tests were used to compare the primary, secondary, and tertiary outcomes between teeth treated with ACM and CM. Cox regression and generalized linear models were used to identify significant predictors of treatment outcomes. All statistical analyses were conducted using SPSS version 25.0 (IBM Corp, Armonk, NY, USA) and GraphPad Prism version 10.0 (GraphPad, La Jola, CA, USA), with *p* < 0.05 considered statistically significant.

## 3. Results

**Figure 1:**
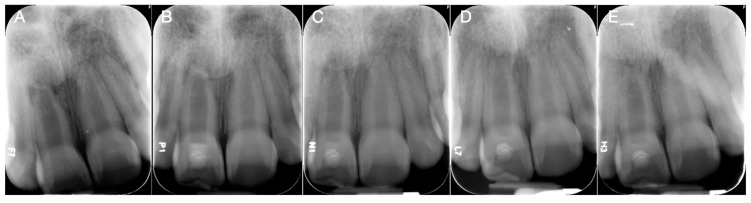
A representative case treated with amnion–chorion membranes. An 11-year-old girl was referred for endodontic treatment of the right maxillary central incisor following an uncomplicated crown fracture. The tooth was diagnosed with pulp necrosis and chronic apical abscess. (**A**) Preoperative radiograph showed the presence of a periapical radiolucency. (**B**) Postoperative periapical radiograph following regenerative endodontic procedures. Dehydrated human-derived amnion–chorion membranes were placed in the root canal along with absorbable collagen matrices. The tooth was restored using Biodentine and composite resin. (**C**) At the 4-month follow-up, the radiograph showed a reduction in size of the periapical radiolucency. (**D**) At the 17-month follow-up, further reduction in the periapical radiolucency was noted. (**E**) At the 25-month follow-up, the radiograph showed complete resolution of the periapical radiolucency.

**Figure 2:**
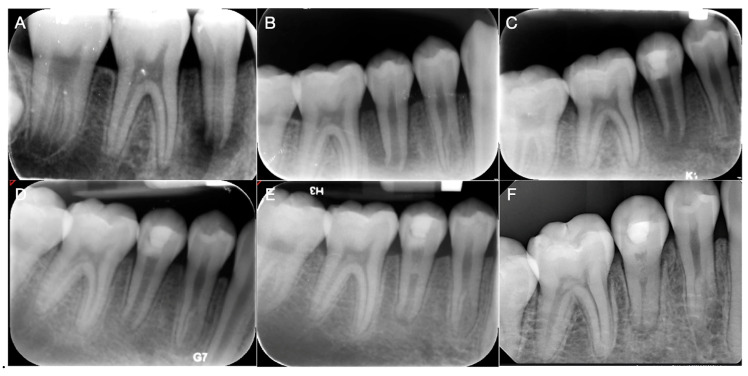
A representative case treated with collagen matrices. An 8-year-old boy was referred for endodontic treatment of the right mandibular second premolar with dens evaginatus. The tooth was diagnosed with pulp necrosis and chronic apical abscess. (**A**,**B**) Preoperative radiograph revealed a periapical radiolucency. (**C**) Postoperative periapical radiograph following regenerative endodontic procedures. Collaplug was placed in the root canal. The tooth was restored using Biodentine and composite resin. (**D**) At the 5-month follow-up, the radiograph showed complete resolution of the periapical radiolucency. (**E**) At the 12-month follow-up, root maturation with mineralized tissue formation in the root canal space was observed. (**F**) At the 46-month follow-up, the radiograph showed complete root maturation along with root canal calcification.

This study included a total of 41 teeth from 38 patients, with an average age of 14.68 ± 7.43 years. Among these teeth, 21 teeth from 20 patients, with an average age of 15.26 ± 7.52 years, had ACM, and 20 teeth from 18 patients, with an average age of 14.11 ± 7.50 years, had CM. The follow-up period ranged from 6 months to 86 months with a mean of 23.23 months and a median of 21 months. Demographic data and clinical characteristics of the study population were summarized in Table 1. Representative cases for ACM and CM are shown in Figure 1 and Figure 2.

### 3.1. Primary Outcome

**Figure 3:**
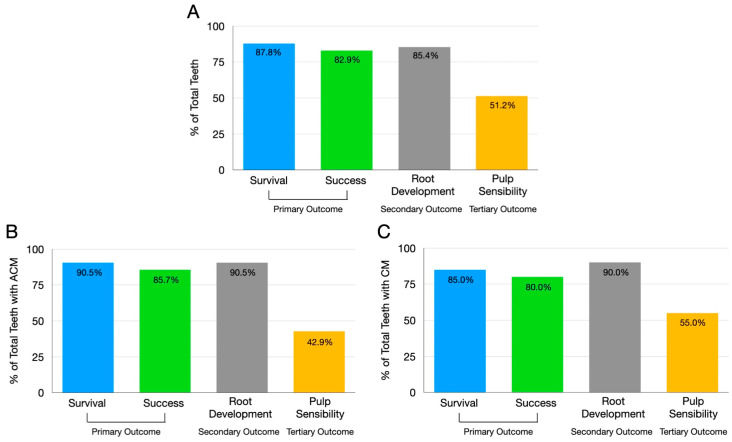
Descriptive statistics of primary (survival and success), secondary (root development), and tertiary (positive response to pulp sensibility testing) outcomes of (**A**) total teeth, (**B**) teeth treated with amnion–chorion membranes, and (**C**) teeth treated with collagen matrices. ACM: amnion–chorion membranes, CM: collagen matrices.

Overall, the total survival rate was 87.8% without clinical symptoms, and the success rate was 82.9% (Figure 3A).

Among the 21 teeth treated with ACM, 90.5% survived without clinical symptoms, and 85.7% demonstrated a successful outcome (Figure 3B). Among the three failures, two cases showed the presence of a sinus tract, and one case showed no clinical signs and symptoms but lacked evidence of periapical healing at the follow-up. All three failures occurred in anterior teeth with trauma as the etiology of pulp necrosis.

For the 20 teeth treated with CM, 85.0% survived without clinical symptoms, and 80.0% achieved a successful outcome (Figure 3C). The four treatment failures comprised a tooth with a crown-root fracture that was extracted, one case showing clinical symptoms, one case with a sinus tract, and one that demonstrated no periapical healing at follow-up. Two failures were associated with trauma, while two occurred in cases with developmental anomalies. Three failed cases involved anterior teeth, and one involved a premolar.

There was no significant difference in survival and success rates between teeth treated with ACM and CM (*p* > 0.05). Cox regression analysis revealed that none of assessed variables including age, gender, tooth type, etiology of pulp necrosis, periapical diagnosis, stage of root development, bioceramic materials, scaffold materials, or permanent restorations were significant predictors of the primary outcome (survival and success) (*p* > 0.05).

### 3.2. Secondary Outcome

Overall, the root development occurred in 85.4% of cases (Figure 3A). The overall increase in RRA was 12.59 ± 12.60%. There was no significant difference in the amount of RRA change between teeth treated with ACM and CM (*p* > 0.05).

**Figure 4:**
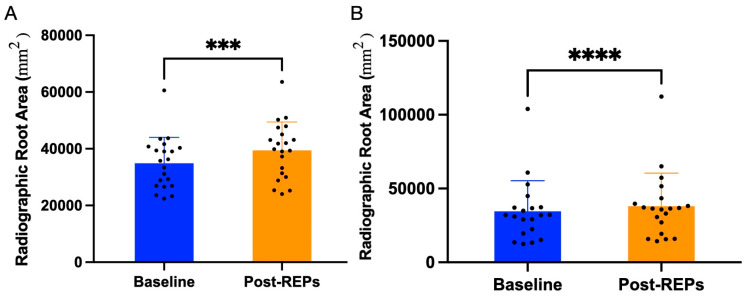
Change in radiographic root area (RRA) following regenerative endodontic procedures (REPs) with amnion–chorion membranes (**A**) and collagen matrices (**B**). A significant increase in RRA was observed at follow-up after REPs compared to the baseline. Post-REPs: post-regenerative endodontic procedures, ***: *p* < 0.001, ****: *p* < 0.0001.

In teeth with ACM, the root development occurred in 90.5% of cases (Figure 3B). The overall increase in RRA was 13.89 ± 13.95%. The median RRA increased from 35,784 mm^2^ (22,445 mm^2^–60,574 mm^2^) at baseline (postoperative) to 39,879 mm^2^ (24,029 mm^2^–63,593 mm^2^) at follow-ups. A statistically significant difference in RRA was observed following REP compared to baseline (*p* < 0.05) (Figure 4A). No root development was observed in three cases that failed.

In teeth with CM, the root development occurred in 90.0% of cases (Figure 3C). The overall increase in RRA was 11.24 ± 11.21%. The median RRA increased from 32,008 mm^2^ (12,431 mm^2^– 103,924 mm^2^) at baseline (postoperative) to 36,462 mm^2^ (14,330 mm^2^–112,317 mm^2^) at follow-ups. A statistically significant difference in RRA was observed following REP compared to baseline (*p* < 0.05) (Figure 4B). Among the failed cases, two showed no root development. However, root development was observed in one case exhibiting a crown-root fracture and in a separate case with clinical symptoms.

After multiple model specifications adjusting for follow-up time, age consistently demonstrated a significant negative association with RRA percentage change when periapical diagnosis was included in the model. In the simplest model including only age and periapical diagnosis, older age was associated with smaller RRA percent change (*p* < 0.05). This effect persisted after adjustment for additional variables including gender, tooth type, etiology of pulp necrosis, stage of root development, bioceramic materials, scaffold materials, or permanent restorations (*p* < 0.05), but was lost when periapical diagnosis was removed from the model. Neither univariate nor multivariable generalized linear models identified any significant predictors of RRA change other than age.

### 3.3. Tertiary Outcome

Among the 41 treated teeth, 51.2% demonstrated a positive response to pulp sensibility testing during follow-up (Figure 3A). There was no significant difference in positive responses to pulp sensibility testing between teeth treated with ACM and CM (*p* > 0.05).

**Figure 5:**
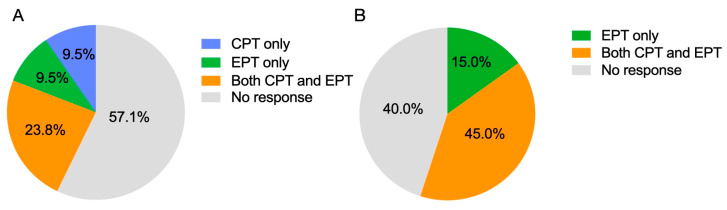
Pulp sensibility outcomes after regenerative endodontic procedures. (**A**) Of the 21 teeth with amnion–chorion membranes, five teeth (23.8%) responded to both cold pulp testing and electric pulp testing, two teeth (9.5%) responded only to electric pulp testing, and another two (9.5%) responded only to cold pulp testing; the remaining 12 teeth (57.1%) were non-responsive to either testing. (**B**) Of the 20 teeth with collagen matrices, eight teeth (45.0%) responded to both cold pulp testing and electric pulp testing, and three teeth (15.0%) responded only to electric pulp testing. CPT: cold pulp testing, EPT: electric pulp testing.

Of the 21 teeth with ACM, 42.9% demonstrated a positive response to pulp sensibility testing during follow-up (Figure 3B). Five cases responded to both CPT and EPT, while two cases responded to CPT only and another two responded to EPT only (Figure 5A). The earliest positive response to CPT was observed at 5.3 ± 4.3 months post-REPs, and for EPT, at 10.9 ± 9.5 months.

Of the 20 teeth with CM, 55.0% demonstrated a positive response to pulp sensibility testing during follow-up (Figure 3C). Eight cases responded to both CPT and EPT, while three responded to EPT only (Figure 5B). The earliest positive response to CPT was observed at 15.9 ± 9.7 months post-REPs, and for EPT, at 18.6 ± 12.4 months.

In the univariate and multivariable regression analyses, no significant associations were observed for age, gender, tooth type, etiology of pulp necrosis, periapical diagnosis, stage of root development, bioceramic materials, scaffold materials, or permanent restorations (*p* > 0.05).

## 4. Discussion

This comparative study represents the first clinical investigation evaluating ACM against CM as commercially available scaffold materials in REPs for immature permanent teeth with pulp necrosis. Our findings demonstrate that ACM effectively promote periapical healing, stimulate root maturation, and restore pulp sensibility, bridging the gap between preclinical research and clinical application. The observed clinical outcomes align with the known biological properties of ACM, supporting in vitro evidence of their ability to enhance dental pulp stem cell migration, promote angiogenesis and mineralization, and suppress proinflammatory cytokines [24]. This translational consistency is further corroborated by an in vivo study in a canine model [23], which showed that ACM outperformed commonly used scaffolds (blood clots and collagen membranes) by generating organized fibrous tissue with functional odontoblast-like cells and reducing periapical inflammation. These findings may position ACM as a biologically active scaffold capable of maintaining the delicate balance between tissue regeneration and immune modulation required for successful REPs.

The study demonstrated favorable clinical outcomes for both scaffold materials, with ACM demonstrating 90.5% survival and 85.7% success rates compared to the 85.0% survival and 80.0% success rates of CM. While these differences were not statistically significant, the results are consistent with existing literature on cell-free REPs. The absence of significant differences between materials provides clinicians with evidence-based options, though the numerical trends may suggest subtle advantages for ACM that could become apparent in larger studies.

The failure patterns provide clinically relevant insights that may guide treatment selection, case management, and prognostic assessment in regenerative endodontics. All three ACM failures occurred specifically in anterior teeth with trauma-related pulp necrosis, suggesting a potential vulnerability in this particular clinical scenario that may be related to the complex pathophysiology of traumatic injuries. Traumatic injuries often involve multiple tissue types, create irregular wound patterns, and may compromise the vascular supply in ways that affect the regenerative potential of different scaffold materials. In contrast, CM failures demonstrated a more diverse etiology profile, including both trauma and developmental causes, distributed across different tooth types including both anterior teeth and premolars. This distribution pattern suggests that CM may be more susceptible to a broader range of clinical variables, though the overall failure rates remained comparable between materials. The heterogeneous failure pattern in the CM group may reflect the different biological mechanisms through which CM support tissue regeneration compared to the more bioactive ACM.

Despite comprehensive regression analysis examining multiple potential predictive factors including age, gender, tooth type, etiology of pulp necrosis, periapical diagnosis, stage of root development, and various treatment variables, no significant predictors emerged among the assessed variables. This absence of clear predictive factors suggests that the therapeutic potential of both materials may be generalizable across diverse patient demographics and tooth characteristics, which has important implications for treatment planning and patient selection criteria.

Root development occurred in 85.4% of cases overall, with comparable rates between ACM (90.5%) and CM (90.0%). Both materials demonstrated statistically significant increases in root surface area (RRA), with ACM showing 13.89 ± 13.95% increase and CM showing 11.24 ± 11.21% increase. While these gains confirm the regenerative potential of both scaffolds to promote dentin deposition and root maturation, the observed RRA increases were relatively modest compared to previous studies reporting changes exceeding 20% [26,27]. This discrepancy can be attributed to specific patient cohort characteristics. Notably, 75.6% of cases involved trauma-induced necrosis, which prior research by Erdogan et al. [28] has associated with significantly lower RRA changes (7.2 ± 13.0%) compared to non-traumatic cases (22.7 ± 24.6%). Additionally, 78.0% of treated teeth were at an advanced root development stage (stage 4), another factor linked to more limited RRA gains [28,29]. These findings highlight the importance of considering etiology and developmental stage when predicting regenerative outcomes.

The identification of age as a significant negative predictor of RRA percentage change represents an important clinical finding. This age-related decline in regenerative capacity is biologically plausible and has practical implications for treatment planning, suggesting that younger patients within the study population may achieve more substantial root development following REPs. Interestingly, the stage of root development was not identified as a significant predictor in the statistical analysis, despite the biological rationale suggesting otherwise. This finding may reflect the specific patient population characteristics or the interaction between multiple variables affecting the regenerative outcome. However, the dependency of the age-related relationship on the inclusion of periapical diagnosis in the statistical model suggests a complex interaction between patient age and the type of periapical pathosis that warrants further investigation in larger, more diverse patient populations.

Pulp sensibility recovery demonstrated notable differences between materials in both timing and response patterns. ACM-treated teeth showed 42.9% positive responses with earlier recovery times (5.3 ± 4.3 months for CPT, 10.9 ± 9.5 months for EPT), while CM-treated teeth achieved 55.0% positive responses but with delayed recovery (15.9 ± 9.7 months and 18.6 ± 12.4 months, respectively). These findings align with reported ranges of literature [30,31,32]. A systematic review and meta-analysis by Li et al. [30] reported that the incidence of positive sensibility response for EPT in necrotic immature permanent teeth after REPs was 25.2%. Clinical studies report varying incidences of positive sensibility responses post-REPs: 11.3% [26], 35.1% [31], and 50% [32].

The earlier sensibility recovery with ACM may reflect its bioactive components, including VEGF and TGF-β1, which facilitate angiogenesis and potentially accelerate neural regeneration [23,24]. However, the overall inconsistency in pulp vitality responses across literature suggests that regenerated tissue may not fully replicate the neurovascular structure of native pulp [4]. Furthermore, our regression analyses identified no statistically significant predictors for pulp sensibility recovery, implying this outcome may result from a complex interaction of biological factors or individual patient responses not captured by the assessed variables. Although positive sensibility responses may indicate functional recovery through neural sprouting or reinnervation rather than complete pulp restoration, future studies should employ more sensitive and specific methods such as laser Doppler flowmetry or pulse oximetry to evaluate true pulp vitality [33].

A distinct aspect of this study was the implementation of a “guided cell homing” approach by using ACM with minimal induction of apical bleeding. While insufficient apical bleeding is a documented cause of failure in REPs [34,35], this study intentionally minimized apical bleeding for specific reasons. Conventional REPs rely on evoked bleeding extending to the cementoenamel junction to indiscriminately recruit cells from the periapical tissues. This process primarily mobilizes resident fibroblasts, osteoblasts, and cementoblasts, with limited recruitment of stem/progenitor cells. Consequently, it often results in the formation of non-physiological, mineralized tissues such as bone- or cementum-like structures within the root canal space, representing an undesirable repair outcome [4,5,23].

ACM leverage their inherent bioactive components to establish a controlled regenerative microenvironment [36]. The membranes naturally contain key growth factors including VEGF, platelet-derived growth factor, TGF-β1, and bFGF alongside anti-inflammatory cytokines (interleukin−4, −6, −8, −10) and tissue inhibitors of metalloproteinases [6,7,8,10,37,38]. These components may act synergistically to create a specialized niche. This specialized niche can perform three critical functions: Firstly, it generates chemotactic gradients that selectively attract beneficial cell populations, such as mesenchymal and hematopoietic stem cells, fibroblasts, and endothelial cells [6,7,10]. Secondly, it regulates the local microenvironment through immunomodulation [6,10,39] and provides sustained angiogenic support [6,7,40,41]. Thirdly, it offers tissue-specific guidance, promoting the formation of vascularized fibrous connective tissue while inhibiting the development of non-physiological mineralized ectopic tissue [18,42,43]. Supporting the efficacy of this approach, in vivo evidence demonstrates that ACM can recruit essential stem cell populations for extended periods up to 28 days [10] and consistently guide the formation of appropriate tissue types [18,43]. By integrating these targeted biological mechanisms, including selective cell homing, microenvironmental regulation, and tissue-specific guidance, the guided cell homing strategy has the potential to overcome the critical limitations of traditional regenerative endodontics, where uncontrolled cell recruitment often leads to non-physiological repair outcomes [5,23]. Although histological confirmation was not performed, this potential is supported by the clinical observations presented in this study, demonstrating that treated teeth exhibited no excessive root canal calcification or pulp canal obliteration and showed a modest gain in RRA, suggesting effective maintenance of adequate root canal space during root development (Figure 1).

In contrast, CM function as a collagen-based scaffold material that offers structural support through its well-characterized three-dimensional matrix architecture [44,45]. This matrix facilitates cell attachment and proliferation through recognized cell–matrix interactions mediated by integrin receptors and other adhesion molecules [46]. The collagen framework provides essential mechanical stability during the early phases of healing and gradually degrades through enzymatic processes as new tissue forms, allowing for organized and guided tissue ingrowth that replaces the scaffold material. However, the degradation time of absorbable CM used in this study is less than 4 weeks, which may not efficiently support the cellular events during the late stage of regeneration [47].

The comparable overall success rates between ACM and CM provide clinicians with evidence-based material options for REPs. The choice between these scaffolds may be guided by specific clinical considerations rather than expected treatment outcomes. The faster sensibility recovery observed with ACM may favor its selection in clinical scenarios where early functional recovery is prioritized, such as in cases involving young patients with high esthetic demands or situations where early confirmation of neural regeneration is clinically important. The bioactive nature of ACM may be particularly advantageous in cases with compromised healing potential or complex clinical presentations. Conversely, the higher overall sensibility response rate achieved with CM, despite delayed timing, may be advantageous in cases where long-term neural regeneration is the primary treatment goal and where the extended regeneration timeline is clinically acceptable.

The limitations of the study include its retrospective design, relatively small sample size, variability in clinical methodologies, reliance on two-dimensional radiography instead of cone beam computed tomography, and the lack of histological analysis. Despite these limitations, the results of this study provide promising preliminary clinical evidence for the use of ACM in REPs. Future prospective, controlled studies with larger cohorts and standardized protocols are needed to validate these findings and explore more comprehensive regenerative outcomes.

## 5. Conclusions

This study provides the first clinical evidence supporting ACM as an effective scaffold for REPs in immature permanent teeth with pulp necrosis, achieving high survival (90.5%) and success (85.7%) rates with faster sensibility recovery compared to CM. While both ACM and CM achieved favorable outcomes, the guided cell homing approach using ACM offers a biologically rational strategy that leverages inherent growth factors and anti-inflammatory properties to potentially overcome limitations of conventional regenerative endodontics. These promising results warrant confirmation by larger, prospective studies with histological validation to comprehensively assess tissue regeneration quality and long-term outcomes.

## Figures and Tables

**Table 1:** Demographic data and clinical characteristics of the study population.

		ACM	CM
Variables	Categories	*n* (%)	*n* (%)
Age	mean ± SD	15.26 ± 7.52 (years)	14.11 ± 7.50 (years)
Gender	Male	12 (60.0)	11 (61.1)
	Female	8 (40.0)	7 (38.9)
Tooth type	Anterior	17 (81.0)	16 (80.0)
	Premolar	4 (19.0)	3 (15.0)
	Molar	0 (0)	1 (5.0)
Etiology	Trauma	17 (81.0)	14 (70.0)
	Caries	4 (19.0)	2 (10.0)
	DA	0(0)	4 (20.0)
Periapical Diagnosis	SAP	8 (38.1)	6 (30.0)
	AAP	8 (38.1)	7 (35.0)
	AAA	3 (14.3)	2 (10.0)
	CAA	2 (9.5)	5 (25.0)
Stage of Root Development (Cvek classification)	Stage 3	2 (9.5)	7 (35.0)
	Stage 4	19 (90.5)	13 (65.0)
Preoperative radiolucency	Yes	21 (100.0)	20 (100.0)
	No	0 (0)	0 (0)
Bioceramic materials	Biodentine	20 (95.2)	14 (70.0)
	BC RRM	1 (4.8)	0 (0)
	MTA	0 (0)	6 (30.0)
Permanent restorations	Composite resin	14 (66.7)	12 (60.0)
	GI	3 (14.3)	4 (20.0)
	RMGI	1 (4.8)	4 (20.0)
	GI + composite resin	1 (4.8)	0 (0)
	RMGI + composite resin	2 (9.5)	0 (0)

ACM: amnion–chorion membranes, CM: collagen matrices, DA: developmental anomaly (dens evaginates, dens invaginatus), SAP: symptomatic apical periodontitis, AAP: asymptomatic apical periodontitis, AAA: acute apical abscess, CAA: chronic apical abscess, BC RRM: Endosequence bioceramic root repair material, MTA: mineral trioxide aggregate, GI: glass ionomer, RMGI: resin-modified glass ionomer, SD: standard deviation.

## Data Availability

The data used for this study were obtained from the electronic health records from Columbia University College of Dental Medicine and include protected health information. Due to institutional policies and patient confidentiality regulations, these data are not publicly available. Access is restricted to approved researchers complying with all applicable ethical and regulatory requirements.
